# Contemporary Survival Selection Fails to Explain Observed Patterns of Phenotypic Divergence Between Suburban and Forest Populations of the Cuban Endemic Lizard, *Anolis homolechis*

**DOI:** 10.3390/biology13121019

**Published:** 2024-12-05

**Authors:** Annabelle Vidal, Frank Cézilly, Roger Pradel

**Affiliations:** 1 Instituto de Ecología y Sistemática, Havana 11900, Cuba; avidalb82@gmail.com; 2 Caribaea Initiative, Le Raizet, 97139 Les Abymes, Guadeloupe, France; 3 Centre d’Écologie Fonctionnelle et Évolutive CEFE, Université de Montpellier, CNRS, EPHE, IRD, CEDEX 5, 34293 Montpellier, Hérault, France; roger.pradel@cefe.cnrs.fr

**Keywords:** body size, capture–mark–recapture, Caribbean fauna, sex-dependent survival, urban ecology

## Abstract

Although urbanization is a major threat to biodiversity, some native species have managed to persist in urban areas. Populations of such species often show phenotypic differences with their rural counterparts. A crucial question is whether such differences result from different selection regimes between habitats. As previous studies showed that suburban and forest populations of *Anolis homolechis* differ in both body size and sex ratio, we tested the effect of urbanization on adult survival in those populations based on a long-term capture–marking–recapture survey and replicated design. We assessed the evidence for directional and stabilizing selection on size in two suburban and two forest populations by modeling apparent adult survival and recapture probability separately. Adult survival did not differ between habitat types. In addition, there was no evidence for size-related differential selection on adult survival between habitats. However, irrespective of habitat, we found significant stabilizing selection on female size, whereas male survival was independent of size. Overall, sex had a significant effect on survival independent of habitat type (monthly survival probability: 0.80 for males vs. up to 0.89 for females of optimal size). We discuss our results in relation to the potential mechanisms involved in the observed phenotypic differentiation of *A. homolechis* and other lizard species in areas transformed by urbanization.

## Simple Summary

The origin of the larger individual body size observed in populations of several lizard species living in urbanized environments compared to individuals in rural populations remains unclear but might be related to differential size-related mortality selection between the two habitats. We tested this hypothesis in the Cuban-endemic lizard *Anolis homolechis* using data from a 20-month capture–mark–recapture survey of two suburban and two forest populations. The relationship between adult survival and body size did not differ between the two habitats. However, adult females of intermediate size showed the highest survival rates, irrespective of habitat, whereas body size had no significant effect on adult male survival. Observed differences in body size between urbanized and natural populations of lizards might, therefore, result from phenotypic plasticity, sexual selection, or size-related selection occurring earlier in life.

## 1. Introduction

Urbanization is a major cause of habitat loss and, consequently, a major threat to biodiversity [1,2,3,4]. In particular, urbanization often leads to biotic homogenization through the replacement of native and/or specialist species with invasive and/or generalist ones [5,6]. Indeed, many native species have been unable to maintain themselves in urbanized environments and have disappeared from previously occupied areas following habitat transformation [7,8,9,10]. However, native species can sometimes persist in urban areas, suggesting some ability to adapt to environmental changes induced by urbanization [11,12,13].

As urbanization is predicted to keep on increasing worldwide in the coming years [14,15], understanding the ecological, behavioral, and physiological processes by which native species can maintain themselves in urban environments [16] is of particular relevance to both evolutionary theory and urban planning [17]. So far, the question has been mainly addressed by comparing phenotypic, ecological, and life history traits between urban and non-urban species [18,19,20,21,22,23] or between urban and non-urban populations of the same species [24,25,26,27,28,29,30]. Over the last 15 years, evidence for phenotypic differences between urban and non-urban populations has been provided for a large range of animals, including both invertebrates and vertebrates (see reviews [1,31,32,33,34,35,36]).

A crucial question is whether such differences result from evolutionary responses to different selection regimes between urban and non-urban habitats or are just the consequences of epigenetic effects, phenotypic plasticity, or non-random dispersal [34,37,38,39]. A few common garden experiments using urban and non-urban populations of invertebrate species have provided convincing evidence for local adaptation to urban environments [40,41,42]. However, only a few studies have quantitatively assessed the direction and intensity of natural and/or sexual selection acting in urban versus natural habitats by studying the fitness consequences of phenotypic variation. Halfwerk et al. [43] found that differences in signaling between urban and forest male túngara frogs, *Physalaemus pustulosus*, were consistent with differences in natural and sexual selection pressures imposed on signalers between the two environments. On the other hand, results from a seven-year study in Southern France failed to support the hypothesis that contemporary reproductive selection explains differences in body size, laying date, and clutch size between urban- and forest-breeding great tits (*Parus major)* [25].

In this context, lizards of the genus *Anolis* provide an excellent opportunity to study morphological and behavioral adaptation to the urban environment. Anoles are one of the best-documented examples of adaptive radiation and evolutionary convergence, with several ecomorphological types having repeatedly evolved in the Greater Antilles [44,45]. Such “ecomorphs” [46] differ in habitat use (vegetation, height, and diameter of perches) and show corresponding differences in morphology, particularly in body size [47,48]. Over the last decade, growing attention has been given to urban populations of anoles (reviewed in [35,37]). Several *Anolis* species can be found to occupy a gradient from natural forests to urban environments [49,50,51]. Various morphological and behavioral differences have been found between urban and non-urban populations in several *Anolis* species [13,52,53,54,55,56,57]. Among them, the larger size of urban anoles compared to their non-urban conspecifics is the most consistent result [35]. However, so far, most studies of urban anoles have concerned invasive species that supposedly face drastic changes in habitat compared to non-invasive, native species [58,59] and have largely focused on males. One exception is the recent studies of the Cuban endemic Habana anole, *A. homolechis*, consisting of a comparison between two suburban populations and two forest populations from different localities [26,60,61]. Results from Vidal et al. [26] showed that suburban individuals of both sexes were larger than forest ones and that sexual size dimorphism was more pronounced in suburban populations. In addition, the sex ratio tended to be male-biased in suburban populations, whereas it did not differ from parity in forest ones. Although various phenomena may explain the observed patterns of variation between suburban and forest populations, one possibility is that the shape/strength of natural selection on body size varies according to sex, habitat, or interaction. Indeed, various factors potentially influencing size-related fitness, such as habitat structure, resource availability, or predation risk, differ between urbanized and natural populations of lizards [62], including anoles [63,64], and may affect the two sexes differently [65].

Here, we directly assessed the evidence for the above hypothesis by addressing the influence of body size on adult survival in the same population of *A. homolechis* studied by Vidal et al. [26]. Although adult survival is a major component of fitness in many species, little information exists about factors affecting adult survival in urban vs. non-urban populations of the same species. Our analysis is based on a long-term capture–mark–recapture (CMR) study allowing us to test hypotheses about variables separately affecting local survival and recapture probability [66]. Only a few studies so far have relied on CMR data to test hypotheses about factors affecting adult survival in native anoles [67,68,69,70,71,72,73], and none of them considered urban populations. Based on results from Vidal et al. [26], we specifically assessed the evidence for a stronger and positive selection for body size in suburban populations of *A. homolechis* compared to forest ones. In addition, we assessed to what extent the influence of sex on adult survival differed between habitats in accordance with observed differences in sex ratio.

## 2. Materials and Methods

### 2.1. Study Sites and Capture Effort

*Anolis homolechis* is a medium-sized ground-trunk anole that is widespread in Cuba, where it lives in filtered sun areas [74]. In the forest, its natural habitat, it is found on edges [74] and cleared paths, whereas in the urbanized environments, it occurs mostly in arborized areas, such as parks and gardens. In both habitats, individuals are active year-round, with a seasonal effect observed only in males, who tend to be more active during the reproductive season [60].

In order to assess the effect of habitat on survival, we relied on a replicate design including two suburban sites and two forested sites. Suburban sites were at the limit of Guanajay City and San José de Las Lajas City, both with similar urban development. The forested sites were located in the relatively well-preserved forests of the Reserve of the Biosphere Sierra del Rosario and the Natural Protected Landscape Escaleras de Jaruco (details and figures in [26,60]).

Data collection took place between January 2018 and August 2019 over a period of 20 months. In each session, two to three persons took part in capturing lizards by noosing from 9:00 to 17:00, corresponding to the period of diurnal activity of the species. Each time, we attempted to capture all detected lizards along fixed transects of about 500 m corresponding to tree-lined streets at the two suburban sites or to natural paths at the two forest sites (see detailed information in [26,60,61]. We initially planned to visit each site once per month. However, due to logistical constraints associated with the socioeconomic situation in Cuba, some sites could not be visited for some months, such that capture effort varied between sites during the course of the study (see Appendix A).

### 2.2. Marking and Data Collection

**Figure 1:**
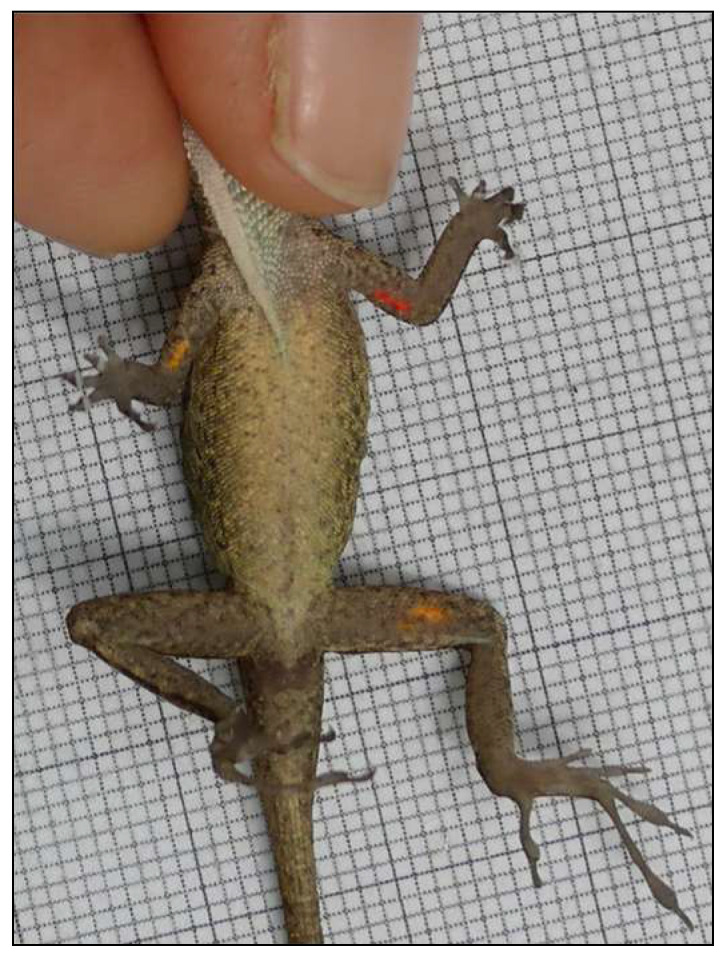
Lizard marked with a combination of two-colored Visible Implant Elastomers (red and orange) in three of the limbs.

Following capture, lizards were kept in individual tissue bags before being marked and processed. We used visible elastomer implants (VIE, Northwest Marine Technology, Inc., Anacortes, WA, USA) to mark individuals. VIE are colorful and fluorescent hypoallergic polymers that can be injected under the ventral skin of the limbs (semi-transparent area), allowing permanent marking [75]. Unique combinations of VIE of six possible colors, three of them implanted in one to three of the four limbs, allowed us to mark hundreds of lizards per site (Figure 1). Captured individuals were measured for body size (snout–vent length, SVL) and classified according to sex and age class. Adult males exhibit a completely developed white-gray dewlap, whereas it is moderately developed in subadult males and almost lacking in females [76]. Males also exhibit a darker coloration than females [70]. Females with an SVL > 35.4 mm were classified as adults, as it was the minimal SVL recorded in a gravid female in the surveyed populations [26]. Marking and measurements were made by the same person (A.V.). Following data collection, all captured anoles were released at the exact location where they had been captured (detailed in [26]).

### 2.3. Statistical Analysis

As our study populations were not closed, we could not differentiate survival from permanent emigration. We, therefore, relied on the Cormack–Jolly–Seber (CJS) model structure for open populations [66] to estimate apparent adult survival probability (*ϕ*) and recapture probability (*p*) separately. For each missing monthly session at any site, we introduced a “virtual” monthly session for which we fixed *p* to zero in the CMR analyses [77,78]. Prior to running survival analyses, we assessed the goodness of fit of the time-dependent CJS model structure to the data with the R2ucare 1.0.2 package [79] of the R Statistical Software 4.2.0 [80]. No heterogeneity was detected.

We constructed alternative models to test different hypotheses about factors influencing *ϕ* and *p*. We considered a null or constant model (.) and the influence of time (*t*), corresponding to each capture session for *p* and to intervals between capture sessions for *ϕ* [77]), sex, body size (SVL at first capture; see Appendix B), habitat (suburban vs. forest), site, and second-order interactions between variables. Based on the observed difference in body size (SVL) between suburban and forest habitats [26], we tested for both directional and stabilizing selection on body size (SVL). If body size is under directional selection, a linear relation is expected between adult survival and the trait, whereas if body size is under stabilizing selection, a quadratic relation is expected between adult survival and the trait. As SVL varied significantly between sexes and habitats [26], we standardized the variable separately for each site and sex (sdSVL: [(SVL—mean SVL)/standard deviation SVL]) after having checked that standardization by sex only or habitat only provided similar results. For recapture probability (*p*), we evaluated the influence of time, sex, body size, and second-order interactions. As we suspected that the probability of detection and capture with a lasso increases with the absolute body size of individuals, body size was included as a linear and non-standardized value of SVL.

Considering all possible combinations of these sources of variation resulted in a total of 348 models. We assessed model fit from Akaike’s Information Criterion for small sample sizes (AICc) [81]. The smallest AICc value indicates the model that provides the best fit to the data. Models that differed by less than 2 AICc units (ΔAICc < 2) from the best model were also considered informative [82]. We further examined whether an interaction would not be limited to one category of a particular effect. For instance, SVL might act on female survival in forests but not in suburban environments. From the eventual best model, we calculated the mean probability of survival between two capture sessions, or monthly survival (*ϕ*), mean annual survival after the first capture (*ϕ*^12^), and the mean lifespan in months as [−1/log(*ϕ*)]. As the estimates were initially obtained on a logit scale (−∞;∞), we transformed them to the scale (0;1) before computing the mean probabilities: *ϕ* = 1/(1 + *e^−logit estimate^*). Recapture probability (*p*) was computed following the same procedure as for survival. Survival analyses were conducted using package RMark 2.2.7 [83] in the R Statistical Software 4.2.0 [76], as an interface to MARK 9.0 [84].

## 3. Results

Overall, we captured and marked 735 adult lizards (435 males, 300 females), of which 17.69% (89 males, 41 females) were recaptured at least once. In suburban sites, we captured 366 individuals and recaptured 61 of them (16.67%), while in forest sites, we captured 369 individuals and recaptured 69 of them (18.70%). The overall rate of recapture did not differ between suburban and forest sites (Chi-square test, *X*^2^ = 0.3647, df = 1, *p* = 0.54599; see Appendix A for capture distribution per site).

According to the best model (Table 1), monthly survival did not vary through time, differed between sexes, and showed a negative quadratic relationship with body size (sdSVL^2^) in females (but not in males), while recapture probability was dependent on time, SVL, and site. There was no evidence for an effect of body size on male survival, whereas we found a significant quadratic effect of body size (sdSVL^2^) on female survival (*ϕ*_females:sdSVL_^2^), suggestive of stabilizing selection. No effect of habitat was retained in the best model. After transformation on the logit scale (Appendix C), male monthly apparent probability of survival (*ϕ*_males_) was estimated to be equal to 0.803 for males, corresponding to an annual probability of survival of 0.072 and a life expectancy of 4.57 months, regardless of habitat. In comparison, the maximum monthly female apparent probability of survival (*ϕ*_females_) was estimated to be equal to 0.893 (for females of optimal size), corresponding to an annual survival probability of 0.258 and a life expectancy of 8.86 months, more than twice that of males.

**Figure 2:**
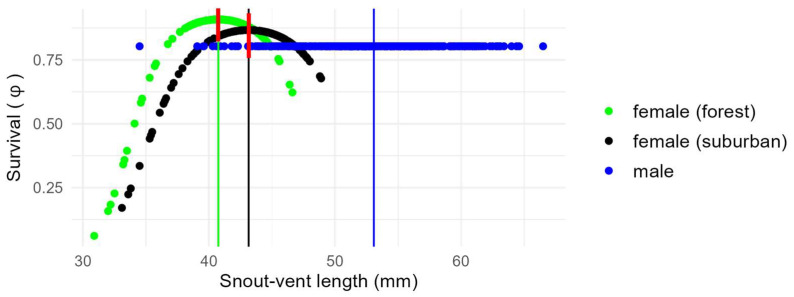
Body size (snout–vent length) effect on the survival probability (*ϕ*) of marked *Anolis homolechis* individuals in suburban and forest habitats based on the second-best model in Table 1. Vertical lines indicate snout–vent length means. Red segments indicate 95% confidence intervals of survival rate for females of mean body size in each habitat, showing a large overlap between the two groups.

The second-best model (ΔAICc = 1.284; Table 1) included an effect of habitat on female survival (*ϕ*_habitat:female_), in addition to the previous effects (Figure 2). However, there was a large overlap in confidence intervals for suburban and forest females (*ϕ*_forest female_: estimator = 0.908, 95% confidence interval [0.823, 0.955]; *ϕ*_suburban female_: estimator = 0.868, 95% CI [0.758, 0.932]), indicating that variation in female survival in relation to habitat was actually negligible. None of the other compared models had similar performance to the best model, including the models that involved hypotheses about habitat-related selection on body size (Table 1).

**Figure 3:**
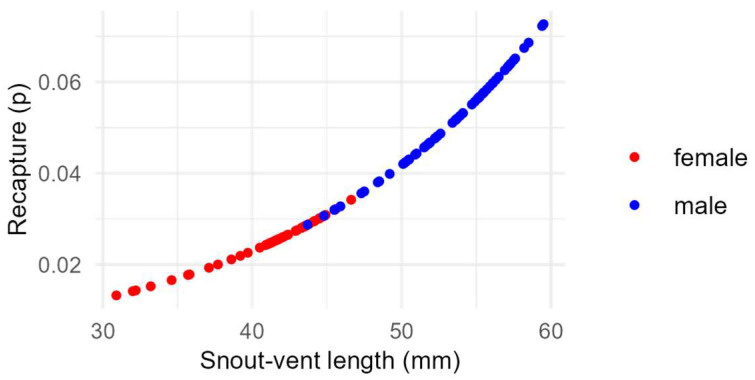
Relationship between the probability of recapture (*p*) and body size (snout–vent length) of *Anolis homolechis*. Data points were computed from the second capture session at Escaleras de Jaruco, according to the best model in Table 1. Each dot corresponds to a single individual. As the effects of both time and sites on recapture rate are additive (on a logit scale), the pattern is similar for all other combinations of site and time (capture session).

**Figure 4:**
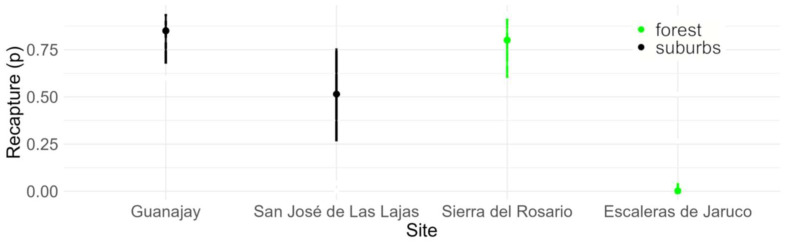
Site effect on recapture probability (*p*) of marked individuals of *Anolis homolechis* from suburban and forest populations based on the best model in Table 1. Values are for an average individual within its habitat-sex category and for the second occasion of capture. Vertical bars indicate 95% confidence intervals.

Recapture probability (*p*_time_; Appendix C) varied between capture sessions with no consistent trend. On the other hand, SVL (*p*_SVL_), but not sex, had a positive and significant effect on recapture (Figure 3). The site effect on recapture probability corresponded to higher values in both Sierra del Rosario (forest) and Guanajay (suburban) compared to Escaleras of Jaruco (forest) and San José de Las Lajas (suburban) (Figure 4).

## 4. Discussion

We investigated to what extent morphological differences observed between suburban and forest populations of an *Anolis* species could be explained by divergent selection between the two habitat types. We found a significant sex effect on adult survival and a negative quadratic effect of body size on female (but not male) survival across habitats. However, our results provided no firm evidence for the hypothesis that differential survival selection on adult body size between forest and suburban habitats is underlying the observed patterns of morphological variation in our studied populations [26]. Similarly, [25] failed to support the hypothesis that contemporary reproductive selection explains observed differences in morphology between urban- and forest-breeding great tits, *Parus major*. However, their study was based upon a single pair of urban and forest populations, with no replications, thus failing to directly assess the effect of urbanization. Here, we investigated survival selection gradients in two suburban and two forest populations in a replicated fashion (allowing us to consider the effect of habitat type independently of that of the study site) and using a large sample size, thus providing statistical robustness to our findings.

We recaptured about 18% of individuals at least once, with no difference between habitats. This estimate is below that reported for some studies on other anole species, with about 35% of individuals recaptured at least once in both *A. mariarum* (n = 1999) [68] and *A. sagrei* (n = 486) [69]. On the other hand, it compares with other studies of anoles in natural habitats, with 14.9% of marked individuals in *A. heterodermus* (n = 458) [73] and 20.4% of marked individuals in *A. desechensis* (n = 452) [72]. However, with 735 marked individuals, our sample size is well above that of those studies and allows a reliable separate estimation of apparent survival and capture probability using CMR methodology [85].

Contrary to several studies [67,68,69], we found an important and positive effect of body size on recapture. The difference could be due to variations between studies in the methods used to capture individuals. Indeed, various methods have been used to sample anoles in the field (see [86]). Otero López [69] reported capturing anoles “by hand”, whereas, unfortunately, no detailed information on the capture method is provided in Andrews and Nichols [67] or Bock et al. [68]. According to our experience in the field, larger individuals were easier to detect and capture with a loop since the weight of the lizard facilitates the closure of the loop around its body and, hence, the capture. Other sources of variation in recapture probability could be related to particular and/or fluctuating conditions both between and within sampling sites. For instance, we noticed that the resident human population of San José de Las Lajas used to prune the vegetation around their homes from time to time, causing drastic changes in the structure of the habitat and a notable decrease in lizards’ detectability the following days. In addition, some heterogeneity in recapture could be due to the fact that captures of anoles involved about twenty different people, of whom some participated only once, while others participated more frequently, and only one (A.V.) participated in all surveys.

Our estimates of adult survival based on CMR data are similar to those reported for *Anolis* species using the same methodology in The Bahamas [71] and Puerto Rico [69] and lower than those reported for a mainland (Colombia) population [68]. Based on our estimates of apparent adult survival, the life expectancy of *A. homolechis* is less than one year in both natural and urbanized habitats. This result agrees with the general pattern proposed by Schoener and Schoener [87] for *Anolis* species of the Greater Antilles and mainland, in contrast to a life expectancy of more than one year for species of the Lesser Antilles and The Bahamas. However, it should be remembered that we could only estimate apparent survival, which, by definition, does not correct for permanent emigration and, therefore, underestimates true survival.

The observed sex-related adult survival in *A. homolechis* is consistent with expectations for polygynous species [88]. Polygynous anole males are more territorial than females [89,90] and defend larger territories [87], therefore being presumably more exposed than females to both injuries during agonistic encounters and predation [91]. However, only limited information is available on factors affecting the demography and survival probability of anoles. In particular, early studies [91,92] were based on analyses of return rates and, thus, did not differentiate between survival and recapture probability, such that our results can only be compared with the few available estimates obtained from the CMR methodology. Similar to our results, Wright et al. [71] found that female *A. sagrei* had a significantly higher monthly survival rate (80%) than males (49%) in The Bahamas. In contrast, Bock et al. [68] found higher annual survival in male *A. mariarum* than in females in northern Colombia. On the other hand, no significant difference in survival between males and females was found in both *A. limifrons* at Barro Colorado Island, Panama [67], and *A. sagrei* in The Bahamas [70]. To what extent variation in survival between sexes directly reflects interspecific differences in the degree of polygyny, as suggested by Schoener and Schoener [87], remains therefore unclear.

Few studies have relied on the CMR methodology to assess the shape of survival selection on body size in anoles. Bock et al. [68] found no evidence for either a linear or a quadratic effect of SVL on survival in males and females of *A. mariarum*, whereas Otero López [69] only tested for the linear effect of body size on survival in *A. cristatellus* and found it to be non-significant. Conversely, Cox and Calsbeek [70] provided evidence for stabilizing the selection of female body size and directional selection of male body size in *A. sagrei*, a trunk-ground anole closely related to *A. homolechis.* We found evidence for stabilizing survival selection on female body size in *A. homolechis* but no evidence for directional selection on male body size, independent of habitat type, despite a more pronounced sexual size dimorphism in suburban populations [26]. One possibility is that increased male size is beneficial in male–male competition for access to females, particularly in the suburban habitat where the sex ratio is male-biased [26], but has no important consequences on survival. Regarding females, body size might be constrained by a higher risk of predation on smaller individuals on the one hand and allometric metabolic costs of reproduction [93] and increased competition with males for trophic resources [94] for larger females on the other hand.

Adaptive morphological evolution depends on selection acting on heritable phenotypic variation [95]. So far, evidence for heritable morphological traits specifically present in urban anoles has been provided only for *A. cristatellus* through a single common garden-rearing experiment in Winchell [13]. In the absence of a clear pattern of survival selection explaining morphological differentiation between suburban and forest populations of *A. homolechis,* Vidal et al. [26] observed differences could then be due to phenotypic plasticity and epigenetic effects [96], sexual selection [97], selective mortality among juveniles [98], or genetic isolation and bottlenecks [98]. One interesting possibility is that artificial light at night in urban and suburban habitats has a strong and positive influence on growth, as recently evidenced in the closely related *A. sagrei* [99]. Indeed, there was no light at night at the forest sites where we conducted our study, whereas some artificial light was provided by a few streetlights or house entrance lights at suburban sites. Future studies in *A. homolechis* should, therefore, examine variation in individual growth and reproduction and assess the heritability of body size success along an urbanization gradient.

## 5. Conclusions

To the best of our knowledge, this is the first CMR study addressing the effect of urbanization on the population biology of an *Anolis* species. The important logistic effort required to collect long-term data on individual phenotypes and fitness probably explains why studies comparing the intensity and direction of natural selection between urban and non-urban habitats remain scarce. Although the results obtained did not allow us to clearly identify the factors underlying morphological differentiation between suburban and forest habitats, our study has resulted in valuable information on adult survival probability and corresponding longevity in *A. homolechis*. It thus provides a first step towards understanding how natural selection can shape urban anole ecology. Similar studies on other *Anolis* species may help to better understand to what extent urbanization affects their population dynamics and demographic parameters. We particularly suggest using the same protocol with other anole species to allow a comparative approach. It would also be particularly interesting to compare ancient and recent colonization of urban environments, for example, by comparing recently urbanized sites with ancient ones to better estimate selection in anole populations with different histories of exposure to urbanized environments.

## Figures and Tables

**Table 1:** Model selection for the estimation of survival in suburban and forest populations of *Anolis homolechis*. Only models from the last round of comparison are presented. The models that describe the initial hypotheses are in bold. Snout–vent length is noted as SVL, standardized snout–vent length as sdSVL, and interactions with colons.

Model		N Parameters	AICc	ΔAICc	Deviance
Survival (*ϕ*)	Recapture (p)				
~male+female:sdSVL^2^+female	*~*time+SVL+site	22	1140.611	0	1095.307
~male+female:sdSVL^2^+habitat:female	*~*time+SVL+site	23	1141.895	1.284	1094.470
~male+female:sdSVL^2^+female	*~*time+SVL+site+sex	23	1142.719	2.108	1095.294
~male+female:sdSVL^2^+habitat:female	*~*time+SVL+site+sex	24	1144.013	3.402	1094.463
**~sex+suburb:sdSVL^2^+forest:sdSVL^2^**	**~time+SVL+site**	23	1145.864	5.253	1098.439
**~sex+suburb:sdSVL+forest:sdSVL^2^**	***~*time+SVL+site**	23	1145.906	5.294	1098.481
**~sex+suburb:sdSVL^2^+forest:sdSVL^2^**	**~time+SVL+site+sex**	24	1147.989	7.378	1098.438
**~sex+suburb:sdSVL+forest:sdSVL^2^**	**~time+SVL+site+sex**	24	1148.012	7.401	1098.462
**~sex+suburb:sdSVL+forest:sdSVL**	**~time+SVL+site**	23	1148.063	7.452	1100.638
**~sex+suburb:sdSVL+forest:sdSVL**	**~time+SVL+site+sex**	24	1150.186	9.575	1100.636

## Data Availability

Data may be obtained by request from the corresponding author.

## Appendix A. Capture Distribution per Site

Capture effort (number of monthly sessions per site) and captures and recaptures of *Anolis homolechis* individuals at suburban and forest sites from January 2018 to August 2019.

		**Number of Sessions**	**Males**		**Females**		**Total**	
			**Captures**	**Recaptures**	**Captures**	**Recaptures**	**Captures**	**Recaptures**
Suburban sites								
	Guanajay	14	123	35	63	11	186	46
	San José de Las Lajas	15	114	11	66	4	180	15
Forest sites								
	Sierra del Rosario	13	136	37	119	22	225	59
	Escaleras de Jaruco	11	62	6	52	4	114	10

## Appendix B. Analysis of Body Size Between Capture Events

In order to both assess whether using SVL at the first capture as a covariate for survival analyses was acceptable and simplify the models, we tested the effect of the SVL at first capture on SVL at last recapture for all recaptured adults. There was a strong and significant correlation between the two values (Pearson’s *r* = 0.90, df = 133, *p* < 0.001). Suspecting that growth between the first and last capture was minimal, we checked whether the slope of the regression between the two SVL measures differed from 1, in which case the two measures would have been equivalent, and it would be acceptable to use only the first SVL measure. We used a simple orthogonal regression that takes into account measurement error on both variables, whereas classic simple regression assumes that only the dependent variable is measured with error [100]. Since the two SVL measures have been taken under the same conditions, this analysis is more appropriate for our data. The first SVL measure had a significant effect on the last one (slope = 1.01, df = 133, *p* < 0.001), and the regression slope was not different from 1 (*t*-test, *p* = 0.99, 95% confidence interval for slope [0.93, 1.10]). This indicates that the SVL at first capture is equivalent to SVL later in life, justifying its use as a measure of body size during the successive events of recapture for survival analyses.

## Appendix C. Survival and Recapture Estimates

Estimates, standard errors, and 95% confidence intervals for survival (*ϕ*) and recapture (*p*) as estimated in the best model of Table 1: *ϕ* dependent on sex and of a quadratic effect of body size (standardized snout–vent length, sdSVL) for females; *p* dependent on time, SVL, and site. Values are given for an individual of average size within its sex-habitat category. Recapture values per session (*p*_time_) are given for the 11 surveys conducted at Escaleras de Jaruco. The quadratic effect of body size for females was estimated on the logit scale as −0.448 (standard error, 0.212; 95% confidence interval, [−0.864, −0.033]). An illustration of this effect is given in Figure 2 for a slightly more general model. Regarding recapture, the detection was higher in all sites other than Escaleras de Jaruco, with constant differences on a logit scale, and increased with size (slope-*p*_SVL_).

**Parameter**		**Estimate**	**Standard Error**	**Confidence Interval**
Survival				
	*ϕ*_male_	0.803	0.534	[0.757; 0.842]
	*ϕ*_female_	0.893	0.577	[0.821; 0.939]
Recapture				
	*p*_time2_	0.038	0.047	[0.003; 0.328]
	*p*_time3_	0	0	_
	*p*_time4_	0.015	0.010	[0.004; 0.054]
	*p*_time7_	0.045	0.024	[0.016; 0.123]
	*p*_time9_	0.025	0.014	[0.008; 0.074]
	*p*_time10_	0.019	0.011	[0.006; 0.057]
	*p*_time11_	0.024	0.013	[0.008; 0.069]
	*p*_time14_	0.033	0.018	[0.011; 0.094]
	*p*_time15_	0.183	0.129	[0.040; 0.548]
	*p*_time18_	0.027	0.016	[0.008; 0.083]
	*p*_time19_	0.035	0.018	[0.012; 0.094]
	*p*_time20_	0.052	0.025	[0.019; 0.130]
	*slope-p*_SVL_ *	0.062	0.019	[0.025; 0.123]
	Δ*p*_Guanajay_ *	1.735	0.511	[0.734; 2.737]
	Δ*p*_San José de Las Lajas_ *	0.056	0.551	[−1.024; 1.136]
	Δ*p*_Sierra del Rosario_ *	1.390	0.503	[0.403; 2.376]
	*p*_intercept_ *	−6.216	1.602	[−9.356; −3.076]
* on a logit scale.				
